# “Best Colleges” and Dr. McKibben

**Published:** 1884-11

**Authors:** P. P. Wells


### "BEST COLLEGES" AND DR. A. B. McKIBBEN.

On page 252, Vol. IV, of HOMŒOPATHIC PHYSICIAN, Dr. McKibben, in her comment on my late stricture on our colleges, says:

> Dr. Wells says, "And yet students but recently from the class-room, at the close of the terms of two of our best (!!!) colleges, have assured me they have heard no word of the philosophy of this science [homœopathic therapeutics] from the first to the last of the course."

Dr. McK. seems disturbed by our use of the word *best* as applied to the colleges the faulty teachings of which it was my purpose to expose. It may be the word was not felicitously chosen to express the meaning we intended. It will be remembered we censured them *all* for a common neglect of what seemed to us a paramount duty—the duty to teach the *philosophy of Homœopathy*. Where *all* were supposed to be alike at fault, it must be apparent it was not our intent, in using this word, to compare the quality of the teaching of these "*two best*" with that of others—but only to say we had this testimony as to two where were found the most numerous faculties and where were gathered the largest classes to be taught. We are glad Dr. McK. has given us the occasion for this explanation, and the more as it gives the opportunity of adding to our former evidence of the neglect complained of, one or two additional facts bearing on the case.

The history of the American Institute, for the last ten years so largely characterized by absence of that which characterizes true Homœopathy, and also largely by adverse resolutions and expressions of silly opinions not in harmony with the spirit or philosophy of that system of therapeutics this Institute was organized to promote, and which has been so largely a disgrace to itself and the system of practical medicine it has been supposed to represent, has been made almost exclusively by the graduates of these colleges. In this history they have presumably given honest expression to the principles, or want of them, they have received from these institutions, which have certified to their fitness and ability to practice a system of healing a knowledge of which they have wholly failed to impart, the fundamental principles of which have never been heard in their lecture-rooms, and of which these accredited graduates before clinical problems show such utter ignorance. Before the facts of this history, are we wrong if for all of disgrace and folly which the Institute has brought on our school we hold our colleges primarily responsible? Who will say that these graduates, so sent out to heal by the use of a system of philosophy they have never learned, and perhaps of which they have never heard,* have not, in making this disgraceful history, *done as well as they knew?* Poor fellows! it was only because they knew no better. They knew no better because they had not been taught.

It would seem there must have been some general principle at the bottom of our college policy to have given us this common experience of ignorance on the part of graduates of that which it is so necessary they should know at the start. The very frank admission of a member of the faculty of one of our homœopathic colleges, when this deficiency in the teaching of his college was pointed out to him and complained of, explains the whole affair, so far as his college is concerned.

" *We do not pretend to make homœopathists of our students. We only make them doctors, and then when they leave, if they wish for Homœopathy they can get it themselves.*"

This is not a truth. They do "*pretend*"—in the *name* of their college—which stands "*Homœopathic* Medical College, of ———." We omit the name of the city which was, and so far as we know is, the home of this very honest and frank professor. They not only "*pretend*" to make their graduates homœopathic physicians, but on every diploma they sell they declare, or are supposed to declare, they have done it. What is their diploma good for else before the homœopathic world? Every diploma then, if this professor, to escape criticism, spoke the truth, is but a lying document to which this frank gentleman presumably has affixed his name. For what do students go to this or any other *Homœopathic* Medical College, rather than to any other kind of a medical college, but that there they may be taught and learn Homœopathy, philosophically and practically? They go for this and for no other reason, and the institution which turns them out not so taught is no better than a fraud. It is of no avail for it to say it "does not pretend" to teach this. It does "pretend," but only does not do it.

We receive with great pleasure and thankfulness the state-

---

*A graduate of last spring of one of our colleges, not "one of the best," recently when questioned as to acquaintance with the contents of the *Organon*, had never seen the book; had heard there was such a book, but as to its contents or purposes had never heard; and as to these confessed utter ignorance. Yet this graduate had the certification of this college to fitness and ability to practice in this community a system of practical medicine, the philosophy of which had never been seen or heard of. Is any condemnation too sharp for neglect and knavery like this? Can we but say—How long shall our patience be abused?

ment that there is *one* college in the country where the *Organon* is taught, and that this is found in St. Louis. Is there another? If there be its whereabouts is unknown.

